# Folding Control in the Path of Type 5 Secretion

**DOI:** 10.3390/toxins13050341

**Published:** 2021-05-11

**Authors:** Nathalie Dautin

**Affiliations:** 1 Laboratoire de Biologie Physico-Chimique des Protéines Membranaires, Université de Paris, LBPC-PM, CNRS, UMR7099, 75005 Paris, France; dautin@ibpc.fr; 2 Institut de Biologie Physico-Chimique, Fondation Edmond de Rothschild pour le Développement de la Recherche Scientifique, 75005 Paris, France

**Keywords:** secretion, folding, autotransporter, type 5 secretion system, intimin, invasin, two-partner secretion, trimeric autotransporter

## Abstract

The type 5 secretion system (T5SS) is one of the more widespread secretion systems in Gram-negative bacteria. Proteins secreted by the T5SS are functionally diverse (toxins, adhesins, enzymes) and include numerous virulence factors. Mechanistically, the T5SS has long been considered the simplest of secretion systems, due to the paucity of proteins required for its functioning. Still, despite more than two decades of study, the exact process by which T5SS substrates attain their final destination and correct conformation is not totally deciphered. Moreover, the recent addition of new sub-families to the T5SS raises additional questions about this secretion mechanism. Central to the understanding of type 5 secretion is the question of protein folding, which needs to be carefully controlled in each of the bacterial cell compartments these proteins cross. Here, the biogenesis of proteins secreted by the Type 5 secretion system is discussed, with a focus on the various factors preventing or promoting protein folding during biogenesis.

## 1. Introduction

In bacteria, protein secretion is essential for numerous processes (nutrients acquisition, pathogenesis, adaptation to the environment, etc.). These organisms have hence developed specific machineries dedicated to this task, 11 of which have been described so far (Type 1 to Type 11 secretion systems: T1SS to T11SS) [1,2,3,4,5,6,7,8,9,10]. These secretion systems are mostly found in didermic bacteria, which harbor, in addition to the cytoplasmic (or inner membrane: IM), an additional outer membrane (OM). However, some of them (T4SS, T7SS) are also present in monodermic bacteria [9,10].

Secretion systems differ by the number, structure, localization, and function of their components; the number and final destination of the secreted substrate proteins (external medium and/or intracellular compartments of other bacteria or eukaryotic cells); and the mechanism of secretion employed (energy requirement, mode of substrate recognition, one or two-step secretion, etc.).

Among secretion systems, the T5SS is one of the more widespread, being found in 62% of Gram-negative bacteria whose genome has been sequenced, with some species even harboring more than 20 different T5SS [11].

Akin to the T2, T8, and T9SS, the T5SS operates in a two-step fashion. The protein to be secreted (the “passenger”) is first exported to the periplasm via the Sec machinery before being translocated through the outer membrane (OM). The translocation across the outer membrane requires a specific, dedicated OM β-barrel protein, called the “translocator” [12]. The passenger and translocator can be part of the same polypeptide chain or produced as two separate proteins (T5bSS). The translocator was initially thought to be the only protein required for OM secretion of the passenger domain. Although it is now clear that it is not the case [13,14], when it comes to the number of specific, necessary factors involved, the T5SS still remains one of the simplest secretion machineries known so far. After OM translocation, the passenger can be released into the surrounding medium or may remain attached to the bacterial cell surface by the translocator.

Proteins secreted by the T5SS are diverse in size, sequence, and function. Although some are produced by environmental species, most of the ones studied so far are produced by pathogenic bacteria and are implicated in the pathogenesis of the producing strain (toxins, adhesins, evasion from the immune system) [15].

**Figure 1:**
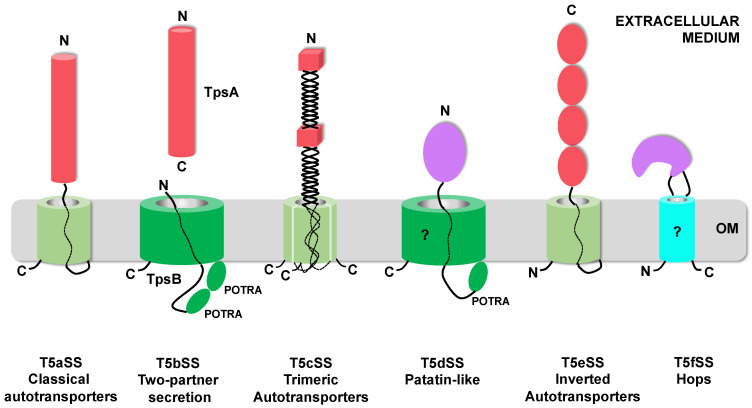
Topological representation of the different T5SS proteins in the outer membrane. The 6 sub-families of the T5SS are represented. Twelve-stranded β-barrels [17,21,24] are represented as pale green cylinders, while BamA-like β-barrels (16-stranded) [19,22] are colored dark green. Hops (*Helicobacter* outer membrane proteins) β-barrel (predicted to be 8-stranded) is colored cyan [32]. A question mark indicates the domains for which no structural data are available. Passenger domains adopting β-rich conformations are depicted as red cylinders for β-helices [16,18], red cubes for β-prisms/β-rolls [20,29], and red ovals for immunoglobulin-like domains [23,28]. Passenger domains adopting α-helical conformation are purple [22,25]. The N and C-termini of each domains are indicated. OM: Outer Membrane.

The T5SS is currently divided into 6 sub-families (T5aSS to T5fSS) based on the three-dimensional structures of their respective translocator and passenger domains and on the organization of these domains relative to each other in the precursor polypeptides (Figure 1). Structural data have been obtained for multiple T5SS proteins (translocator and/or passenger domains) [16,17,18,19,20,21,22,23,24,25,26]. The only ones not structurally characterized yet are the translocator domains of the T5dSS and T5fSS.

The T5aSS comprises the “classical” autotransporters. They are produced as single polypeptide chains comprising an N-terminal passenger domain and a C-terminal translocator domain, which forms a 12-stranded β-barrel in the OM. Both domains are connected by a linker that traverses the OM through the pore formed by the β-barrel (Figure 1) [17]. In this sub-family, the passenger can vary widely in size and usually, but not exclusively, adopts a β-helix fold [16,25,27]. In the T5eSS (“inverted” autotransporters), the domains are inverted. The translocator domain, which also forms a 12-stranded β-barrel in the OM, is N-terminal, while the passenger domain, which adopts an elongated structure composed of multiple, independent, immunoglobulin-like domains, is C-terminal [23,24,28]. The T5cSS (“trimeric” autotransporters) also relies on a 12-stranded β-barrel for outer membrane translocation. However, as their name indicates, they are trimeric: 3 subunits, each contributing 4 strands, assemble to form the barrel. The sequence coding for these 4 β-strands is found at the C-terminus of the passenger domain. The three secreted passenger domains form an intertwined, elongated structure composed of a succession of α-helical coiled-coil and β-rich (β-prisms, β-rolls) domains, linked by connector regions [20,21,29].

In the T5bSS, the translocator is a larger, 16-stranded β-barrel, belonging to the BamA family and possessing 2 periplasmic POTRA (polypeptide-transport-associated) domains [19]. Upstream from the 2 POTRA domains is an α-helix that occludes the translocator pore when in the resting state and moves to the periplasm during substrate secretion [19]. The translocator, also called the TpsB protein, is produced independently from the passenger (the TpsA protein), hence the name: “two-partner secretion” (Tps) for this sub-family. As in the T5aSS, the substrates secreted by the T5bSS are predicted to fold mostly as long β-helices to which additional functional domains can be added [18,30].

In the T5dSS, a single polypeptide chain comprises an N-terminal passenger domain, followed by a single POTRA domain and a C-terminal domain predicted to form a 16-stranded β-barrel homologous to TpsB proteins (Figure 1) [31]. In that case, the secreted substrates identified so far adopt a globular, compact, α/β hydrolase fold typical of patatin-like lipases [22]. The last addition to the T5SS, the T5fSS sub-family, includes Hops (*Helicobacter* outer membrane proteins), which are predicted to form an 8-stranded β-barrel in the OM, needed for the translocation of 15–110 kDa α-helical passenger domains. Here however, the passenger domain is not connected to one of the barrel termini, but instead constitutes one of the extracellular loops of this β-barrel (Figure 1) [32].

Despite this variety in structure and topology, it is currently assumed that all proteins belonging to the T5SS have a rather similar mode of biogenesis. After synthesis, they are kept unfolded in the cytoplasm until they are exported through the inner membrane via the Sec translocon. Once in the periplasm, the translocator domain is targeted to the OM by periplasmic chaperones and folded and inserted into the OM by the BAM (β-barrel assembly machinery) complex [33,34]. There, in a still controversial mechanism, the β-barrel allows the translocation of the passenger domain to the cell surface, where it adopts its final, functional conformation.

During their journey from their site of synthesis to the external medium, the folding of the different domains of T5SS proteins need to be carefully and temporally controlled. Here, what is currently known about this control and the factors involved in each cellular compartment is discussed.

## 2. Staying Unfolded in the Cytoplasm

### 2.1. Targeting Pathways to the Inner Membrane

**Figure 2:**
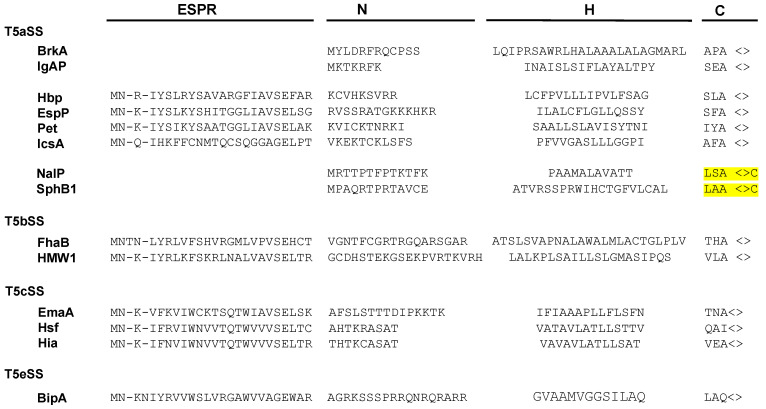
Signal sequences of T5SS proteins. Although they represent the majority of signal peptides found in T5SS, only two examples of canonical signal sequences, harboring the N, H, and C regions are shown (BrkA and IgAP). Multiple extended signal sequences, which comprise the conserved N-terminal ESPR (Extented Signal Peptide Region), are shown, with examples from each sub-family. Two signal peptides from lipidated autotransporters (NalP and SphB1) are also depicted, with the lipobox highlighted in yellow.

Numerous secreted proteins are identified by the presence of a 20–30 amino acid long N-terminal signal sequence (or signal peptide) which is cleaved during export through the inner membrane. Signal sequences show little sequence conservation but have a common organization. They are composed of three regions: a N-terminal region (N) which comprises a majority of basic residues, a H region predominantly composed of hydrophobic amino acids, and a C region, which includes the cleavage site (Figure 2) [35].

Signal sequences are recognized by molecular chaperones and targeting factors as they emerge from the ribosome. This ensures that proteins are delivered to the correct translocon and kept in the appropriate conformation for export. Indeed, whereas the TAT (twin-arginine translocation) translocase exports only folded proteins, the Sec translocon transports unfolded proteins across the inner membrane. All T5SS proteins harbor N-terminal signal peptides. None of them have been identified as crossing the inner membrane via the TAT translocon. Hence, as for other Sec-dependent presecretory proteins, the folding of T5SS proteins in the cytoplasm must be prevented and the proteins kept in a soluble, secretion-competent conformation to ensure proper export and to prevent aggregation and degradation.

**Figure 3:**
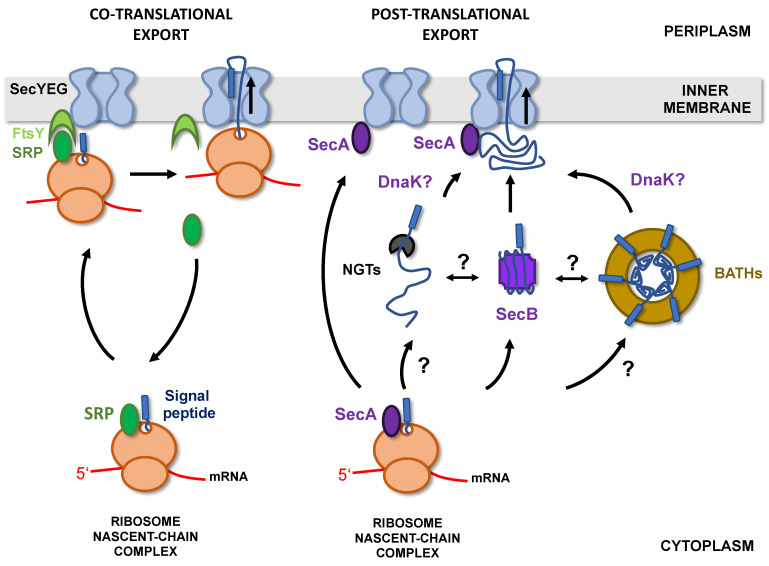
T5SS proteins targeting to the inner membrane. T5SS proteins can be targeted to the inner membrane co-translationally (**left**) or post-translationally (**right**). Co-translational export required the recognition, by SRP (signal recognition particle), of nascent chains as they emerge from the ribosome. The ribosome-nascent chain complex (RNC) is then targeted to the Sec translocon (SecYEG) via SRP recognition of its receptor FtsY. Export then proceeds co-translationally. In the case of post-translational targeting, proteins are released in the cytoplasm, where they need to be kept unfolded and soluble until export through SecYEG. This is achieved by interactions with chaperones such as SecB or DnaK. Some T5SS are also glycosylated in the cytoplasm by 2 classes of glycosyltransferases: the NGTs (HMW1C-like N-glycosyltransferases) and the BAHTs (bacterial autotransporter heptosyltransferases) which potentially recognize partially folded substrates.

In bacteria, there are two major pathways allowing protein targeting to the Sec translocon [36]. The SRP/FtsY pathway relies on a ribonucleoprotein (SRP for signal recognition particle) that recognizes hydrophobic sequences as they emerge from the ribosome and targets the ribosome-nascent chain complex (RNC) to the SecYEG-bound FtsY receptor. The nascent chains are then transferred to the Sec translocon and exported through the inner membrane (IM) co-translationally (Figure 3) [37].

Co-translational export, by limiting protein exposure to the cytoplasmic environment, is an efficient way to prevent premature folding or aggregation of secreted proteins in the cytoplasm. However, the SRP/FtsY pathway is mostly dedicated to the targeting of inner membrane proteins and only 2% of secreted proteins are addressed to the membrane by this route [38]. Most presecretory proteins are instead directed to the inner membrane post-translationally, in a process that depends on the essential ATPase SecA and cytoplasmic chaperones such as SecB, Trigger Factor (TF), or DnaK [39].

In the case of the T5SS, targeting to the inner membrane has been exclusively studied for a limited number of proteins that contain atypical signal sequences, longer than 50 residues. These T5SS-specific signal peptides, which are found in about 10% of T5SS proteins, have been identified in all T5SS subfamilies, except Type 5f and 5d. They consist of a C-terminal part resembling a canonical signal sequence and a conserved 25-residue N-terminal extension, called the “extended signal peptide region” (ESPR) (Pfam: PF13018) (Figure 2) [40].

While the ESPR region alone cannot act as a signal sequence [41,42], the C-terminal part of the extended signal peptide is usually sufficient to target proteins to the inner membrane and ensure their export [43,44,45]. One exception is the passenger domain of Vta9, a trimeric autotransporter from *Haemophilus parasuis* which, when expressed in *E. coli*, is not exported in the absence of ESPR [46].

The elongated signal sequences of the classical *E. coli* autotransporters (ATs) EspP, Pet, and IcsA allow post-translational, SecB-dependent targeting to the inner membrane [45,47,48,49] However, the deletion of ESPR in EspP leads to co-translational, SRP-dependent export [45,49]. It was therefore initially proposed that the ESPR region could inhibit the interaction between the C-terminal part of the signal sequence and SRP. However, it was subsequently shown that EspP in fact binds SRP immediately after its synthesis but is then excluded from the SRP/FtsY targeting pathway by a TF-dependent mechanism [50,51]. A similar result was obtained with FhaB, a protein secreted by the T5bSS in *Bordetella pertussis*, which interacts transiently with SRP immediately after its synthesis but is then targeted post-translationally to the inner membrane via the SecA/SecB pathway. In that case, deletion of the ESPR extension has no influence on the addressing pathway [52]. In contrast to EspP, Pet, or FhaB, Hbp, a classical AT produced by pathogenic strains of *E. coli*, is addressed to the inner membrane co-translationally, in the presence or absence of the ESPR region [43,53]. Finally, the trimeric autotransporter EmaA from *Aggregatibacter actinomycetemcomitans* was shown to be targeted to the inner membrane in an SRP-independent, SecB-dependent manner, but the role of ESPR in targeting was not studied. In the absence of SecB, DnaK could partially compensate for the defect in EmaA secretion [54]. DnaK was also implicated in the secretion of two classical ATs (IcsA and SepA) [55] and proposed to interact with a third (Ag43) [56]. Whether its role is ubiquitous in T5SS or limited to T5SS proteins harboring an ESPR extension is currently unknown.

Overall, these studies suggest that the ESPR region has only a little role in determining the targeting pathway of T5SS proteins to the inner membrane. However, they indicate that depending on the T5SS protein, export across the inner membrane can be post- or co-translational.

When post-translational targeting occurs, chaperones (SecB, DnaK) appear essential to maintain T5SS proteins in a secretion-competent state until they reach the IM. Whether these chaperones interact with specific domains (passenger and/or translocator domains) of T5SS proteins or if multiple chaperones can bind simultaneously to these usually large proteins (>100 kDa) is not yet known.

So far, Hbp is the only T5SS protein reported to be co-translationally exported [43,53]. Initially, it was proposed that proteins exported co-translationally fold too rapidly in the cytoplasm to be addressed post-translationally. Indeed, several of these proteins were not efficiently secreted when fused to post-translational signal peptides [57]. In contrast, Hbp could be efficiently secreted when fused to the post-translational signal peptide from PhoE, provided that SecB was present [43]. This indicates that co-translational export is not a prerequisite for Hbp secretion.

Because the targeting pathway of only a handful of T5SS proteins has been established (<10), it remains to determine how prevalent co-translational export is in the T5SS.

Certain classical autotransporters (T5aSS) have a signal sequence characteristic of lipoproteins, comprising, at the cleavage site, a “lipobox” ([LVI]-[ASTVI]-[GAS]-C) (Figure 2). Lipoproteins are proteins modified in the periplasmic leaflet of the IM by the addition of lipids on the invariant cysteine of the lipobox [58]. In *E. coli*, export of the two lipoproteins: Lpp (murein lipoprotein) and BRP (Bacteriocin Release Protein) is independent of SecB and instead requires SRP together with YidC, a universal membrane protein insertase that functions either together or independently from the Sec translocon [59,60,61]. It is thus possible that, similarly to these 2 lipoproteins, lipidated autotransporters are exported co-translationally.

It is currently unknown whether co-translational export is strictly necessary for a subset of T5SS proteins exhibiting fast folding kinetics in the cytoplasm. In fact, folding kinetics have not been compared between co- and post-translationally exported T5SS proteins. However, studies performed on the passenger domains of two classical ATs (Ag43 and Pertactin (Prn), both most likely exported post-translationally), have shown that folding is extremely slow in vitro (t_1/2_ ~hrs to days) [62,63]. This observation is in line with a recent study from Loos et al. [38], who proposed that in *E. coli* K12, mature domains of secreted proteins, independently from signal peptides, have evolved enhanced flexibility and a tendency to fold more slowly compared with cytoplasmic proteins. Secreted proteins indeed appear enriched in small and polar amino acids and have fewer aggregation- prone regions and hydrophobic patches. These characteristics were hypothesized to limit premature cytoplasmic folding, independently of chaperones [38]. Interestingly, mutations introducing small hydrophobic clusters in the N-terminal disordered region of Prn caused conformational ensemble contraction and increased aggregation in vitro together with secretion defects in vivo when introduced in the full-length protein. Although it is most likely that in the case of T5SS, these properties influence translocation of the passenger domain across the OM [64], whether they also influence the mode of export through the inner membrane or the need for interaction with cytoplasmic holdases, before export, has not been tested.

Finally, the targeting pathway/mode of export and role of cytoplasmic chaperones remains to be determined for T5SS proteins lacking an ESPR sequence and for members of the newest T5SS subfamilies (T5dSS, T5eSS, and T5fSS).

### 2.2. Cytoplasmic Glycosylation and Folding

The passenger domain of several members of the T5SS, including classical ATs (T5aSS) [65,66,67,68], proteins secreted by the two-partner secretion system (T5bSS TpsA proteins) [69,70], and trimeric ATs (T5cSS) [71,72], have been shown to be glycosylated at multiple sites (e.g., 31 for the TpsA HMW1A and 18 for the classical autotransporter Ag43). Two different mechanisms of glycosylation have been identified, and in both cases, modification occurs in the cytoplasm, before export.

HMW1A/2A and EtpA are proteins secreted by the T5bSS in *H. influenzae* and enterotoxigenic *E. coli* respectively. They are glycosylated by dedicated glycosyltransferases (HMW1C/2C and EtpC), the genes of which are co-transcribed with the respective *tpsA* and *tpsB* genes. These homologous glycosyltransferases are interchangeable [73] and belong to a family of enzymes (the “NGTs” for HMW1C-like N-glycosyltransferases) able to transfer single hexoses from UDP-hexoses to asparagine on the acceptor NXS/T sequons (where X ≠ Pro). In some species, genes encoding NGTs are found alone, without associated T5bSS, and can modify classical or trimeric autotransporters, as well as proteins not related to T5SS [71,74]. Whether these cytoplasmic glycosyltransferases recognize their substrates co-translationally, as they emerge from the ribosome, similarly to peptide deformylase and methionine aminopeptidase [75,76] or post-translationally, after complete synthesis, is unknown. In addition, the state of folding of the acceptor protein when modification occurs has not been determined. On the one hand, the recognition of a sequence motif, together with the ability of NGTs to modify short peptides in vitro [74,77] suggest that these enzymes do not recognize a structural motif in their targets. However, a recent study coupling mass spectrometry and proteomics showed that NGTs act in a semi-processive manner, with a preference for sequons that are exposed on the surface of the acceptor protein, suggesting a post-translational modification of partially folded substrates [78].

The second mechanism of T5SS protein glycosylation involves an unrelated family of glycosyltransferase, the BAHTs (bacterial autotransporter heptosyltransferases), which transfer heptoses on serine or threonine residues of target proteins [66,67,79]. The crystal structure of the glycosylated passenger domain of TibA (TibA_55–350_), a classical autotransporter, indicates that the sugars are aligned on the surface of the protein, mostly attached to the loops connecting the β-strands of the β-helix [80].

BAHTs, which are also commutable, are either co-transcribed with their target or located elsewhere on the chromosome [67,68,81]. These enzymes use ADP-glycero-β-D-manno-heptose from the lipopolysaccharide biogenesis pathway as a sugar donor, and require iron for activity [68,70]. *E. coli* TibC, the only BAHT that has been crystallized so far, forms a large (578 kDa) ring-shaped dodecamer (external diameter 145 Å, height 72 Å) with the catalytic site of each subunit pointing towards the central pore of the ring (internal diameter 110 Å). The complex between TibA_55–350_ and TibC was purified and analyzed by cryo-electron microscopy. The structure obtained (at 8.9 Å resolution) shows six molecules of TibA_55–350_ inserted in the pore of the TibC ring, in an elongated, spiral shaft conformation fitting well with the crystal structure of folded TibA_55–350_ [80]. These results, which suggest that BAHTs modify folded (or partially folded) substrates, are consistent with the previously proposed idea that these enzymes recognize a structural motif rather than a consensus sequence [82] and the position of the modified residues, on the surface of the target protein, in loops connecting the β-strands [68].

Whatever the glycosylation mechanism (NGTs- or BATHs-mediated), a number of questions remain about how the glycosylation reaction is spatially and temporally coordinated with protein targeting to the inner membrane (Figure 3). In particular, how, and at what point during/after synthesis, are proteins handed over to glycosyltransferases instead of being directly exported without modifications? How is specificity established? Are glycosyltransferases interacting with cytoplasmic chaperones and targeting factors? How are the glycosylation-competent conformations of passenger domains (apparently partially folded) attained and maintained? What happens to the linked β-domain (which is not glycosylated) during the glycosylation steps? In particular, in the case of BAHTs substrates, is the passenger domain folding before or upon interaction with the BAHTs ring? Can BAHTs modify any β-helix-forming passenger domain? Finally, once glycosylated, how are these domains released, unfolded, and addressed to the inner membrane for export?

Canonical chaperones/targeting factors could transfer T5SS proteins to glycosyltransferases, remain bound to them while they are being modified, or address them to the membrane after modification. Whether NGT or BAHT glycosyltransferases directly participate in inner membrane targeting of glycosylated T5SS is unknown. Because the HMW1A level in the cytoplasm was strongly decreased in the absence of the NGT HMW1C [70], this enzyme was hypothesized to act as a chaperone for HMW1A prior to export, eventually protecting it from proteolytic degradation [83]. However, glycosylation itself is known to increase stability and proteolytic resistance of proteins, including the BAHT-modified ATs Ag43 and AIDA-I [63,79]. Glycosylation was also found to increase the rate of Ag43 folding in vitro [63].

Aah, the BAHT necessary for AIDA-I glycosylation, was proposed to be membrane-associated [84], but an eventual interaction with SecYEG was not tested. Importantly, glycosyltransferases are not required for the secretion of Ag43, AIDA-I, or HMW1A [63,70,79] and although glycosylated T5SS proteins often possess an ESPR sequence, neither this sequence nor the entire signal peptide are required for glycosylation [82]. As previously mentioned, SecB and DnaK are important for the secretion of EmaA, an *N*-glycosylated, trimeric AT [54]. Ag43, an *O*-glycosylated, classical AT from *E. coli*, was isolated as part of the DnaK interactome [56]. In that case, DnaK, which has an unfolding activity, might be necessary for unfolding the passenger domain after it has been glycosylated in the BAHT ring. Alternatively, glycosylated autotransporters might interact with unidentified, specific chaperones.

## 3. Periplasmic Transit

T5SS proteins exported by the Sec translocon emerge in the periplasm, with their N-terminus first, in an unfolded conformation. The translocator domains must then reach the outer membrane, in which they are inserted/folded by the BAM (β-barrel assembly machinery) or TAM (translocation and assembly module) machineries [85,86,87]. Meanwhile, the long passenger domains must be prevented from aggregation and degradation and maintained in a conformation competent for proper translocation across the OM. In most subfamilies, the passenger domain is fused to the translocator domain and hence targeted with it to the OM. In the case of the T5bSS, the passenger (TpsA protein) is independently targeted to the OM, where it is recognized by the already inserted translocator (TpsB protein).

### 3.1. Transient Anchoring to the Inner Membrane

Although the ESPR extension has apparently no role in determining the targeting pathway of T5SS proteins to the inner membrane, it appears to be important for proper periplasmic transit. Szabady et al. [41] have indeed shown that in the absence of ESPR, the classical autotransporter EspP is properly exported through the inner membrane but tends to misfold in the periplasm, leading to decreased passenger domain translocation across the OM. Because overexpression of a fusion between EspP signal sequence and the maltose binding protein impairs the translocation of various envelope proteins across the inner membrane, it was proposed that the ESPR extension interacts with the Sec translocon to reduce overall rates of IM transport. This sequence would hence slow down export of T5SS proteins and prevent the passenger domain from folding compactly in the periplasm [41]. Similarly, the extended signal sequence from FHA (secreted by the T5bSS) significantly decreases the rate of export of beta-lactamase when it replaces its native signal sequence [52]. However, although the presence of the ESPR sequence also delays the inner membrane translocation of Pet [48], this classical autotransporter is still efficiently secreted when the ESPR is deleted [44]. In contrast, the extended signal peptide of the trimeric autotransporter EmaA is important for proper assembly of the monomers into a functional adhesin [42]. Finally, an ESPR sequence has also been found in at least one protein secreted by the T5eSS (the “inverted” autotransporters) (Figure 2) [88]. However, the role of ESPR in this family, where the first domain to emerge in the periplasm is the translocator, has not been determined.

How exactly the ESPR sequence is slowing down export via Sec has not been determined. In the case of Hbp, the extended signal sequence interacts with YidC [61,89]. In the absence of YidC, Hbp accumulates in the periplasm in a form which is susceptible to degradation by the DegP protease [89]. The secretion of another classical AT, EspC, is also affected in the absence of YidC [89], suggesting that ESPR could delay export by interacting with YidC. However, neither an ESPR-dependent export delay of Hbp and EspC nor an interaction between ESPR and YidC has been demonstrated [89]. In addition, the role of YidC in the secretion of EspP, FHA, or EmaA has not been tested, whereas the secretion of IcsA, another AT harboring an extended signal sequence (Figure 2), is YidC-independent [47]. Alternatively, the ESPR extension could also delay export by preventing the association between Sec and a factor necessary to clear the translocon from its substrates. One potential candidate is PpiD, an inner membrane-anchored periplasmic chaperone, which interacts with SecY at the same position as YidC and improves translocation efficiency [90,91].

The second question is how does slowing down export across the inner membrane prevent passenger domain misfolding in the periplasm and/or facilitate OM translocation through the OM? Initially, it was proposed that delaying export could facilitate interactions between T5SS proteins emerging in the periplasm and soluble periplasmic chaperones [41]. Indeed, slowing down export of Prn in *E. coli* (by replacing its native post-translational signal peptide by a co-translational or ESPR-containing one) suppresses the requirement of the DegP chaperone for cell viability [92], suggesting that export rate directly influences the conformation of the passenger domain in the periplasm. Alternatively, transient anchoring to the inner membrane could also help recruit inner membrane or outer membrane factors important for periplasmic transit or OM translocation. For instance, an ESPR-containing trimeric autotransporter (SadA) depends on an inner membrane trimeric lipoprotein (SadB) for proper export to the surface [93]. Although not tested, a transient anchoring of SadA to the inner membrane could facilitate SadB recruitment. Similarly, recruitment of OM insertion machineries, such as TAM or BAM, could be favored by longer retention times of T5SS proteins at the Sec translocon. TAM is indeed an intermembrane-spanning complex, with the inner membrane component (TamB) contacting the outer membrane insertase TamA via an elongated taco shell-shaped periplasmic domain [94]. The involvement of TAM in the secretion of certain T5SS proteins has been demonstrated [87,95,96] and the inner membrane component, TamB, appears essential for the proper folding of the passenger domain at the cell surface [97]. Whether TamB captures T5SS proteins as they exit the Sec translocon and targets them to TamA without periplasmic release is not clear yet. No interaction between TamB and Sec has been demonstrated, nor a tripartite complex consisting of TamAB with a substrate isolated [87,97]. However, an ESPR-containing AT (Ag43) is able to interact with both TamA and TamB in vivo [97].

In contrast with TAM, the BAM machinery was found to associate with the Sec translocon to form a trans-periplasmic complex. Here, it is still unclear if this complex is formed constitutively or upon export of substrates [98,99,100]. Additionally, the eventual substrate specificity of this complex or its potential role in T5SS has not been determined.

### 3.2. Role of Periplasmic Factors in Maintaining Secretion-Competent Conformation

In the periplasm of Gram-negative bacteria, a network of well-described ATP-independent quality control factors is necessary for the biogenesis of outer membrane proteins. This network includes chaperones/holdases such as SurA (survival protein A), FkpA (FK506-binding protein A), or Skp (seventeen kilodalton protein), as well as proteins with dual chaperone/protease activity like DegP. In physiological conditions, none of these factors are essential due to their redundant function and overlapping substrate specificity [101].

The role of these quality control factors is variable depending on the T5SS protein. Studies have shown that in *E. coli*, *S. flexneri*, and *Yersinia*, the lack of SurA affects passenger domains secretion for classical (EspP, Hbp, IcsA) [102,103,104], and inverted (EPEC Intimin and *Yersinia* Invasin) autotransporters [105,106,107,108]. In these cases, the effect on β-barrel insertion in the OM differs from one protein to another. Direct interactions between SurA and Hbp or EspP have been demonstrated in vivo by cross-linking approaches or yeast two-hybrid (YTH) experiments [103,109] and in vitro, by surface plasmon resonance (SPR) [103]. These studies indicate that SurA binds not only to the β-barrel but also to the passenger domain of T5aSS proteins. Similarly, Skp interacts with both the passenger of EspP and Pet and with the β-barrel domain of EspP and NalP [110,111,112]. However, deletion of *skp* was found to affect the biogenesis of EspP and IcsA, but not that of Pet, Hbp, Intimin, or Invasin [102,103,104,105,108,112,113]. The passenger domain of EspP was found to also interact with FkpA by SPR, but the effect of deleting *fkpA* on EspP biogenesis was not tested [114]. Finally, in strains lacking *degP*, the secretion of EspP and IcsA was defective while Hbp translocation was unaffected [104]. Interactions between DegP and the passenger or translocator domains of EspP were revealed by SPR and YTH [103]. As previously mentioned, contrary to Pet or Hbp, expression of *Bordetella* Prn was lethal in an *E. coli degP* strain, while the lack of either *skp* or *fkpA* did not cause lethality or defect in Prn secretion [92,113].

Importantly, UV-dependent cross-linking experiments coupled with pulse-chase analysis have shown that interactions between periplasmic chaperones and their substrates are sequential; indeed, EspP first interacts with Skp, then with SurA and the components of the BAM machinery (interactions with FkpA or DegP were not reported in this study) [110]. Skp would hence act early after the protein emerges in the periplasm, while SurA would target the protein to the BAM complex for folding and insertion in the OM. Altogether, these studies point to a central role of SurA in T5aSS protein transit through the periplasm, while the other quality control factors would only have a secondary role in maintaining the passenger domain in a form compatible with secretion and/or in degrading the misfolded, potentially toxic intermediates. The essential role of SurA in T5aSS biogenesis is corroborated by the fact that, in vitro, SurA and the BAM complex are necessary and sufficient to insert and fold autotransporters into proteoliposomes or nanodisks [115,116]. The dependency on other factors in vivo could be governed by the folding properties, rate of inner membrane export, expression level, or OM insertion machinery used by individual T5SS proteins.

**Figure 4:**
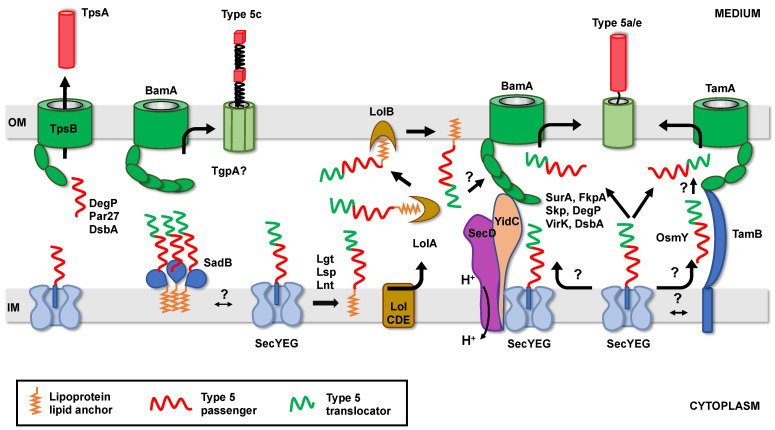
T5SS proteins transit through the periplasm. T5SS proteins exit the Sec translocon in an unfolded conformation, with their N-terminus first. Some may be transiently associated with the Sec translocon, via their N-terminal ESPR sequence. Once released into the periplasm, they interact with chaperones and targeting factors that address them to the BAM and/or the TAM complex for insertion and folding in the outer membrane. For clarity, the BamBCDE subunits are not represented. A portion of T5SS proteins is acylated, after export, by the dedicated lipoprotein modification pathway consisting of 3 enzymes: Lgt, Lsp and Lnt. Although not proven, lipidated T5SS proteins might be addressed to the OM via the Lol system, before OM insertion and folding by BAM or TAM. IM: Inner Membrane, OM: Outer Membrane.

Interestingly, apart from the factors described above, which have a general role in outer membrane proteins biogenesis, putative chaperones have also been identified as implicated only in T5SS biogenesis. Such a putative periplasmic chaperone protein, VirK, (conserved in *Enterobacteriaceae*), interacts with the unfolded passenger domain of Pet (but not with its β-barrel) and is necessary for its secretion [112]. A role for VirK in the biogenesis of IcsA was also suggested but not thoroughly investigated [117]. This putative chaperone does not seem to have a general role in the biogenesis of OMPs (porins), however its exact substrate specificity has not been determined [112]. Another recently identified periplasmic protein (OsmY) is necessary for the biogenesis of several classical autotransporters (Ag43, TibA, and EheA) in *E. coli*. In vitro studies indicate that this protein would act by promoting the folding and stabilization of the translocator domain but not that of the passenger domain. OsmY appears specific to SAATs (Self Associating AutoTransporters, which includes glycosylated ATs), its absence having no influence on the biogenesis of EspP. Although not confirmed, it was proposed that OsmY would be part of an OM-addressing path parallel to the SurA/BAM canonical path (Figure 4). Indeed, the ATs Ag43 and EhaA are substrates of the TAM insertion machinery and OsmY interacts with TamB [118]. Whether VirK or OsmY have a role in other T5SS sub-families, outside the “classical” autotransporters, remains to be established.

Importantly, the studies described above have been mostly performed in *E. coli* or close relatives. However, the role of these chaperones in the wider bacterial community may vary significantly. In *N. meningitidis* for instance, SurA, Skp, and DegP appear to have a minor role in OMPs biogenesis or AT secretion. Indeed, although an interaction has been demonstrated in vitro between *E. coli* Skp and NalP (an AT from *N. meningitidis*) [111], in vivo, IgAP, another classical AT of *Neisseria*, does not show any synthesis defect in the absence of *skp*, *surA*, or *degP* [119].

In the T5bSS, the TpsA protein is released in the periplasm independently from the TpsB protein, which, like other OMPs, probably relies on the previously described network to reach the OM, at least in *E. coli*. Whether this network is also necessary to keep TpsA proteins in a secretion-competent conformation until they reach their cognate TpsB translocator is mostly unknown. In the absence of their cognate TpsB proteins, certain TpsA (ShlA, OtpA) accumulate in the periplasm, while others (FHA, HMW1A, CupB5) are degraded by DegP [120,121,122,123,124]. In *Bordetella pertussis*, Par27, a chaperone with peptidyl prolyl isomerase activity, was identified as interacting with the TpsA FHA. In vitro, both Par27 and DegP prevent FHA aggregation and degradation. However, FHA secretion was not affected in a *B. pertussis par27* strain [125] but was decreased in a *degP* strain [120]. Additionally, none of these proteins were required for FHA translocation in an in vitro reconstituted system [126].

The periplasmic transit of trimeric autotransporters (T5cSS) appears to significantly differ from other T5 subfamilies. Although mutants of YadA (a *Yersinia* trimeric AT) defective in OM translocation were degraded by DegP [127,128], yeast two-hybrid experiments failed to detect interactions between YadA and SurA or Skp. When YadA was expressed in yeast, it could fully integrate as functional trimers in the OM of mitochondria and the level of assembly was significantly increased in the presence of Skp, but not of SurA [129]. However, the biogenesis of YadA was not affected in *Y. enterocolitica* strains where the *skp*, *surA*, or *degP* genes have been deleted [108]. Interestingly, few trimeric ATs genes are found to be co-transcribed with genes encoding potential periplasmic chaperones. In *S. typhimurium*, the export and folding of the trimeric AT SadA is affected when SadB, a trimeric lipoprotein associated to the inner membrane, is absent. Although an interaction between SadA and SadB has not been detected, it was proposed that SadB could support the trimerization of unfolded SadA and improve translocation across the OM by increasing the retention time of the unfolded passenger at the inner membrane [93]. In *Acinetobacter* sp. Tol 5, a gene encoding a periplasmic protein is co-transcribed with the trimeric AT AtaA. When this protein (TpgA: trimeric autotransporter and peptidoglycan-associated protein A) is absent, the level of AtaA displayed at the cell surface decreased. TpgA has no similarity with SadB and is monomeric. It interacts both with the peptidoglycan and with AtaA at the outer membrane [130]. Operons similar to *sadBA* and *aatA-tpgA* were found in various Gram-negative bacteria, however the exact mechanism of action of these two putative chaperones, together with their substrate specificity, is currently unknown. In particular, it remains to be determined if they could also act on other trimeric ATs or other OMPs.

In addition to chaperones and proteases, enzymes catalyzing the formation and isomerization of disulfide bonds (DsbA/DsbC), are also found in the periplasm of Gram-negative bacteria. Some T5SS passenger domains indeed harbor native disulfide bonds, suggesting they interact with the Dsb system while transiting through the periplasm. However, this has been verified only in a few cases. DsbA was found essential for the formation of a disulfide bridge located in the C-terminal lectin-like domain of Intimin. In the absence of this disulfide bond, Intimin passenger domain is still secreted, but in a conformation susceptible to proteases [105]. Similarly, the Dsb system was required for the formation of the disulfide bridge located in the passenger of IcsA [131]. In some TpsA proteins, a disulfide bond is formed at the C-terminus of the protein, in a domain that remains periplasmic after translocation. The role of Dsb in the formation of this disulfide bond, which is necessary for the protein anchoring to the cell surface [132], has not been addressed. In general, T5SS proteins have a low cysteine content and when present, the cysteines are only a few residues apart. In these conditions, disulfide bond formation should only generate small loops in the unfolded precursor that should not interfere with OM translocation [113]. In this context, it is interesting to note that a family of chlamydial classical ATs, the Pmp (polymorphic membrane protein) can display up to 18 cysteines in their passenger domain [133]. How these T5SS proteins are handled in the periplasm has not been examined.

As previously mentioned, some T5SS proteins are lipoproteins. In Gram-negative bacteria, lipoproteins are tri-acylated, after export to the periplasm, by the consecutive action of 3 enzymes: Lgt, Lsp, and Lnt (Figure 4) [58]. Lipoproteins destined to the OM interact with the LolCDE complex, which transfers them to LolA, a soluble periplasmic protein. LolA subsequently handles the substrate to LolB (an OM lipoprotein) for insertion of the lipid moiety in the inner leaflet of the OM [58]. Some lipoproteins can be further translocated across the OM [134]. In the case of lipidated T5SS proteins, the final destination of the lipid anchor (inner or outer leaflet of the OM) and hence the topology of the corresponding proteins in the OM, have not been determined. In addition, the role of the Lol system in their biogenesis, and its eventual interplay with the periplasmic factor previously described, has not been studied.

Finally, in contrast with the previously described member of the T5SS, the role of periplasmic factors in the biogenesis and folding control of T5dSS and T5fSS proteins have not been investigated.

## 4. Translocator Domain Insertion and Folding in the Outer Membrane

### 4.1. Role of the BAM Complex

In 2003, a conserved system essential for the insertion and folding of β-barrel proteins in the outer membrane of Gram-negative bacteria was discovered [33]. Since then, the now-called BAM (β-barrel assembly machinery) complex has been extensively studied both biochemically and structurally [33,85,86].

The BAM machinery is a hetero-oligomeric complex composed of an outer membrane protein (BamA), and a variable number of lipoproteins (4 in *E. coli*: BamB-E). BamA is essential and conserved in Gram-negative bacteria. The C-terminal domain of BamA forms a 16-stranded β-barrel in the OM, while its N-terminal domain consists of five POTRA domains located in the periplasm [135,136,137,138,139]. Multiple T5SS have been shown to depend on the BAM complex for biogenesis, which is expected since these proteins contain β-barrel-forming domains. BAM is indeed necessary for the secretion of classical [33,104,109,110,140,141], inverted [105], and trimeric [128,142] ATs, but its role in TpsB protein biogenesis, or T5d and T5f autotransporters, has not been studied. Interactions between T5SS proteins and various components of the Bam complex (BamA, BamB, and BamD) have also been demonstrated [104,109,110].

Although the mechanism by which BAM inserts and folds β-barrels in the OM is not completely deciphered [34,85,86,143], structural studies have indicated that BamA can adopt multiple conformations, with its N- and C-terminal β-strands pairing either via 8 (closed barrel) or only 2 (open barrel) hydrogen bonds [135,136,137,138,139]. It was hence proposed that the substrate terminal β-strands could pair with the BamA lateral seam to initiate β-sheet formation in the OM. This led to a “budding” model in which the substrate β-barrel forms a hybrid barrel with BamA and buds out in the membrane once folding is complete [85,86,135,143]. This model is supported by the recent observation, by cryo-electron microscopy, of such a hybrid barrel formed between BamA and a substrate [144]. However, this complex represents a late-stage intermediate in the assembly of the substrate and does not permit to determine whether the hairpins of the substrate β-barrel are formed before or upon interaction with BamA. Indeed, site-specific cross-linking studies performed on classical and trimeric ATs indicate that the β-barrels of these proteins already acquire a certain degree of folding in the periplasm, prior to their insertion in the OM [145,146]. In these cases, the periplasmic proto-barrels would be transferred onto the opening of BamA by a “swing” mechanism [147]. 

How the b-barrels are finally released into the OM is less clear [86].

### 4.2. Role of the TAM Complex

More recently, another complex (TAM: Translocation and Assembly Module) has been identified as important for the secretion of certain T5SS proteins [87]. TamA is a homologue of BamA, also forming a 16-stranded β-barrel in the outer membrane with a lateral opening, but harboring only 3 POTRA domains [148,149].

TAM is necessary for the secretion of some classical autotransporters such as Ag43 and EhaA in *E. coli* and p1121 in *Citrobacter* [95], as well as some inverted ATs (Intimin, FdeC) [150], but has apparently no role in trimeric ATs assembly (YadA, EibD, SadA) [151]. Its role in other T5SS subfamilies has not been established. It is not clear yet what properties of β-barrels make them substrates for TAM instead of BAM. Specificity might not be that stringent since Ag43, a TAM substrate, is efficiently processed by BAM in an in vitro reconstituted system [152] and Intimin has been found to depend on BAM for secretion in vivo [105]. Although the structural and functional homologies between TamA and BamA suggest that these two proteins may have similar mechanisms to insert ATs in the OM (“budding” model), this needs to be established. The exact role of TamB in this process also needs to be determined. It was recently shown that TamA POTRA domains, upon substrate binding, extend into the periplasm. Because these domains interact with the relatively rigid TamB, the resulting pressure applied on TamA intramembrane domain was proposed to induce a local distortion of the OM that could promote β-barrel assembly [87,96].

## 5. Passenger Domain Secretion and Folding in the Extracellular Space

The mechanism by which T5SS proteins secrete their passenger domain across the OM has been the subject of numerous debates and will only be briefly discussed here as several excellent reviews have covered these subjects recently [30,153,154]. In the case of the two-partner secretion system (T5bSS), the TpsB protein forms a channel in the OM through which the TpsA protein is secreted. TpsB recognizes its periplasmic, unfolded substrate via an interaction between its POTRA domains and the N-terminal domain of the TpsA protein. What remains unclear is the orientation of the substrate (N-terminus outside or inside) as it emerges in the extracellular space and what is the energy source driving translocation [30,154]. In the case of autotransporters, the main questions are the nature of the channel translocating the passenger, and, as for T5bSS, the energy source driving translocation through the OM [153]. The question of the energy source might have found a partial answer with the recent demonstration of a role for the proton motive force (PMF) in the assembly of outer membrane proteins by BAM. However, a direct link between the PMF and the translocation of ATs passenger domains across the OM needs to be established [98]. It also remains to be determined whether all ATs passenger domains are translocated through their own β-barrel or through a hybrid barrel formed with BamA during OM folding and insertion [13]. What is generally accepted, however, is that the passenger domains emerge unfolded in the extracellular space, the region closer to the barrel (i.e., the passenger C-terminus in the T5aSS and T5cSS, the N-terminus in the T5eSS) forming a hairpin through the membrane [62,155]. These passenger domains then need to rapidly reach their functional conformation at the cell surface. In a few classical ATs, an “autochaperone” domain has been identified at the C-terminus of the passenger domain. This domain promotes folding of the full passenger domain whether provided in cis or in trans (as a separate polypeptide) [12,156]. In some ATs, a domain of increased stability (“stable core”) can also be found at the C-terminus of the passenger domain [12]. It was proposed that this stable core could nucleate folding in the extracellular space, which would then occur in a vectorial fashion from the C- to the N-terminus and drive translocation of the rest of the passenger through the OM. These autochaperone domains and stable cores are however absent from passenger domains adopting an α-helical conformation [156]. Nevertheless, the structure of at least one α-helical passenger domain (*Rickettsia* Sca) suggests that it could also fold vectorially [27]. Whether this is also true in other sub-families where passenger domains are mostly α-helical (T5dSS, T5fSS) is unknown. No autochaperone/stable core domain were identified in inverted autotransporters. In the case of Intimin, deletion of the membrane-proximal domain does not affect folding or secretion of the rest of the passenger, suggesting that in that case, each Immunoglobulin-like domain folds individually in a sequential manner [155]. Finally, folding of the passenger domain in the extracellular space might also be assisted by the β-barrel itself. Indeed, Yuan et al. [157] showed that the extracellular loop 5 of EspP and Pet β-barrel forms a β-hairpin at the cell surface, which is essential for the folding of the passenger domain. A mechanism was proposed in which the β-barrel L5 β-hairpin would promote folding of the passenger domain by β-nucleation. This mechanism could be widespread in the T5SS. Indeed, an external β-sheet region of the TpsB protein FhaC was also found to be important for the secretion of its cognate TpsA [158]. Finally, in the X-ray structure of Intimin and Invasin, the extracellular loops L4 and L5 were found to form a small β-sheet with the extremity of the linker [24]. However, the role of these loops in passenger domain folding and/or translocation across the OM has not been tested.

## 6. Conclusions

The T5SS proteins, initially thought to be able to drive their own secretion, in fact require numerous accessory factors for proper travel from their site of synthesis to their final destination. While most of these accessory factors have a general role in the biogenesis of secreted proteins, others appear specific to the T5SS. The requirement for some of these accessory factors varies between sub-families, but also within members of the same sub-type, which indicates that findings obtained with a single T5SS protein cannot be generalized neither to the entire T5SS nor to one of its sub-families. Importantly, studies performed so far have been limited to only a few bacterial species (mostly *E. coli* and close relatives). Thus, the eventual role of the various accessory factors identified so far needs to be established in more distant bacteria (when present).

## Data Availability

Not applicable.
